# Sequence Alterations of I(Ks) Potassium Channel Genes in Kazakhstani Patients with Atrial Fibrillation

**DOI:** 10.5195/cajgh.2014.147

**Published:** 2014-12-12

**Authors:** Ainur Akilzhanova, Saule Rakhimova, Zhannur Abilova, Omirbek Nuralinov, Gulzhaina Rashbayeva, Ayan Abdrakhmanov, Mahabbat Bekbosynova

**Affiliations:** 1 Center for Life Sciences, Nazarbayev University, Astana, Kazakhstan; 2 National Scientific Cardiac Surgery Center, Astana, Kazakhstan

**Keywords:** atrial fibrillation, delayed rectifier potassium channel, genetic analysis

## Abstract

**Introduction:**

Atrial fibrillation (AF) is the most common sustained arrhythmia, and it results in significant morbidity and mortality. However, the pathogenesis of AF remains unclear to date. Recently, more pieces of evidence indicated that AF is a multifactorial disease resulting from the interaction between environmental factors and genetics. Recent studies suggest that genetic mutation of the slow delayed rectifier potassium channel (I(Ks)) may underlie AF.

**Objective:**

To investigate sequence alterations of I(Ks) potassium channel genes KCNQ1, KCNE1 and KCNE2 in Kazakhstani patients with atrial fibrillation.

**Methods:**

Genomic DNA of 69 cases with atrial fibrillation and 27 relatives were analyzed for mutations in all protein-coding exons and their flanking splice site regions of the genes KCNQ1 (NM_000218.2 and NM_181798.1), KCNE1 (NM_000219.2), and KCNE2 (NM_172201.1) using bidirectional sequencing on the ABI 3730xL DNA Analyzer (Applied Biosystems, Foster City, CA, USA).

**Results:**

In total, a disease-causing mutation was identified in 39 of the 69 (56.5%) index cases. Of these, altered sequence variants in the KCNQ1 gene accounted for 14.5% of the mutations, whereas a KCNE1 mutation accounted for 43.5% of the mutations and KCNE2 mutation accounted for 1.4% of the mutations. The majority of the distinct mutations were found in a single case (80%), whereas 20% of the mutations were observed more than once. We found two sequence variants in KCNQ1 exon 13 (S546S G1638A) and exon 16 (Y662Y, C1986T) in ten patients (14.5%). In KCNE1 gene in exon 3 mutation, S59G A280G was observed in 30 of 69 patients (43.5%) and KCNE2 exon 2 T10K C29A in 1 patient (1.4%). Genetic cascade screening of 27 relatives to the 69 index cases with an identified mutation revealed 26.9% mutation carriers who were at risk of cardiac events such as syncope or sudden unexpected death.

**Conclusion:**

In this cohort of Kazakhstani index cases with AF, a disease-causing mutation was identified in 56.5 % of the referred patients. Further screening of mutations in other genes encoding cardiac ion channels is needed to clarify possible disease causing and founder mutations in Kazakhstani atrial fibrillation patients.

